# On the bounds of degree-based topological indices of the Cartesian product of *F*-sum of connected graphs

**DOI:** 10.1186/s13660-017-1579-5

**Published:** 2017-12-13

**Authors:** Muhammad Imran, Shakila Baby, Hafiz Muhammad Afzal Siddiqui, Muhammad Kashif Shafiq

**Affiliations:** 1 Department of Mathematical Sciences, United Arab Emirates University, P. O. Box 15551, Al Ain, United Arab Emirates; 2 Department of Mathematics, School of Natural Sciences (SNS), National University of Sciences and Technology (NUST), Sector H-12, Islamabad, Pakistan; 3 Department of Mathematics, Government College University Faisalabad (GCUF), Jhang Road, Faisalabad, Punjab Pakistan; 4 Department of Mathematics, COMSATS Institute of Information Technology (CIIT), Defence Road, Off Raiwind Road, Lahore, Punjab Pakistan

**Keywords:** 92E10, 05C90, *F*-sum, Cartesian product, ABC-index, Zagreb index, GA-index, *F*-index

## Abstract

Topological indices are the mathematical tools that correlate the chemical structure with various physical properties, chemical reactivity or biological activity numerically. A topological index is a function having a set of graphs as its domain and a set of real numbers as its range. In QSAR/QSPR study, a prediction about the bioactivity of chemical compounds is made on the basis of physico-chemical properties and topological indices such as Zagreb, Randić and multiple Zagreb indices. In this paper, we determine the lower and upper bounds of Zagreb indices, the atom-bond connectivity (ABC) index, multiple Zagreb indices, the geometric-arithmetic (GA) index, the forgotten topological index and the Narumi-Katayama index for the Cartesian product of *F*-sum of connected graphs by using combinatorial inequalities.

## Introduction and preliminary results

We consider *G* as a simple, connected and finite graph with a vertex set $V(G)= \{u_{1}, u_{2}, u_{3}, \ldots , u_{n}\}$, an edge set $E(G)= \{e_{1}, e_{2}, e_{3}, \ldots , e_{m}\}$, the *order* of $G=\vert V(G)\vert =n$ and the size of $G=\vert E(G)\vert =m$. An *edge* $e\in E(G)$ with end vertices $u_{i}$ and $u_{j}$ is denoted by $u_{i}u_{j}$. The number of edges having *u* as an end vertex is called the *degree* of *u* in *G* and is denoted by $\operatorname{deg}_{G}(u)$. The minimum and maximum degrees of graph *G* are denoted by $\delta_{G}$ and $\Delta_{G}$, respectively. $P_{n}$ and $C_{n}$ are used for *path* and *cycle* with order *n*, respectively.

The branch of chemistry in which we discuss and predict the chemical structure by using mathematical tools without referring to quantum mechanics is called *mathematical chemistry* [1, 2]. The branch of mathematical chemistry which applies graph theory to mathematical modeling of chemical phenomena is known as *chemical graph theory* [2]. This theory has a remarkable role in the development of chemical sciences.

The *Zagreb* indices are the first degree-based structure descriptors [3, 4]. The terms $\sum_{v \in V(G)} [\operatorname{deg}_{G}(v) ]^{2}$, $\sum_{uv \in E(G)}\operatorname{deg}_{G}(u)\operatorname{deg}_{G}(v)$ and $\sum_{v \in V(G)} [\operatorname{deg}_{G}(v) ]^{3}$ first appeared in the topological formula for total *π*-energy of conjugated molecules that was derived in 1972 by Gutman and Trinajstić [3]. Ten years later, Balaban et al. included

$$M_{1}(G)= \sum_{v \in V(G)} \bigl[\operatorname{deg}_{G}(v) \bigr]^{2}= \sum_{uv \in E(G)} \bigl[\operatorname{deg}_{G}(u)+\operatorname{deg}_{G}(v) \bigr] $$

and

$$M_{2}(G)= \sum_{uv \in E(G)}\operatorname{deg}_{G}(u)\operatorname{deg}_{G}(v) $$

among topological indices and named them ‘Zagreb group indices’ [5], which was later on abbreviated to ‘Zagreb indices’, and now $M_{1}(G)$ and $M_{2}(G)$ are called the first and second *Zagreb* indices. Afterwards these indices were used as branching indices [6]. Later on, the Zagreb indices found applications in QSPR and QSAR studies [1, 7]. These indices have been used to study molecular complexity, chirality, *ZE*-isomorphism and hetero-systems. Chemical applications and mathematical properties of Zagreb indices can be studied from [8–10].

Narumi and Katayama studied the degree product of a graph G for the first time in 1984. The *Narumi-Katayama* index proposed by Narumi and Katayama [11] is defined as follows:

$$\operatorname{NK}(G)= \prod_{v\in V(G)}\operatorname{deg}_{G}(v). $$

Estrada et al. [12] introduced the atom-bond connectivity index defined as follows:

$$\operatorname{ABC}(G)= \sum_{uv \in E(G)}\sqrt{\frac{\operatorname{deg}_{G}(u) + \operatorname{deg}_{G}(v)-2}{\operatorname{deg}_{G}(u) \operatorname{deg}_{G}(v)}}. $$

It has been applied up till now to study the stability of alkanes and the strain energy of cycloalkanes [12, 13]. The ABC-index can be used for modeling thermodynamic properties of organic chemical compounds. The ABC-index happens to be the only topological index for which theoretical, quantum-theory-based, foundation and justification have been found.

The first geometric-arithmetic connectivity index or simply geometric-arithmetic (GA) index of a connected graph *G* was introduced by Vukičević et al. in 2009 [14] and is defined as follows:

$$\operatorname{GA}(G)= \sum_{uv \in E(G)}{\frac{2 \sqrt{\operatorname{deg}_{G}(u) \operatorname{deg}_{G}(v)}}{\operatorname{deg}_{G}(u) + \operatorname{deg}_{G}(v)}}. $$

The augmented Zagreb index proposed by Furtula et al. in 2010 [15] is defined as follows:

$$\operatorname{AZI}(G)= \sum_{uv \in E(G)} \biggl[\frac{\operatorname{deg}_{G}(u) \operatorname{deg}_{G}(v)}{\operatorname{deg}_{G}(u)+\operatorname{deg}_{G}(v)-2} \biggr]^{3}. $$

This graph invariant is a valuable predictive index in the study of the heat of formation in octanes and heptanes [15].

The first multiple Zagreb index was introduced by Ghorbani and Azimi in 2012 [16] and defined as follows:

$$\operatorname{PM}_{1}(G)= \prod_{uv\in V(G)} \bigl[\operatorname{deg}_{G}(u)+\operatorname{deg}_{G}(v) \bigr]= \prod _{v\in V(G)} \bigl[\operatorname{deg}_{G}(v) \bigr]^{2}. $$

Clearly, the first multiple Zagreb index is the square of Narumi-Katayama index.

The third Zagreb index was introduced by Shirdel in 2013 [17] and defined as follows:

$$M_{3}(G)= \sum_{uv \in E(G)} \bigl[\operatorname{deg}_{G}(u)+\operatorname{deg}_{G}(v) \bigr]^{2}. $$

Furtula and Gutman showed that the term $\sum_{v \in V(G)} [\operatorname{deg}_{G}(v) ]^{3}$ has a very promising application potential [18]. They called it the forgotten topological index or shortly the *F*-index, and it is defined as follows:

$$F(G)= \sum_{v \in V(G)} \bigl[\operatorname{deg}_{G}(v) \bigr]^{3}= \sum_{uv \in E(G)} \bigl[ \bigl(\operatorname{deg}_{G}(u) \bigr)^{2}+ \bigl(\operatorname{deg}_{G}(v) \bigr)^{2} \bigr]. $$

They proved that the linear combination $M_{1}+ \lambda F$ yields a highly accurate mathematical model of certain physico-chemical properties of alkanes [18].

Clearly, this index is a combination of second and third *Zagreb* indices, i.e.,

$$F(G)=M_{3}(G)-2 M_{2}(G). $$

The Cartesian product is an important method to construct a bigger graph and plays an important role in the design and analysis of networks [19]. The Cartesian product of the graphs *G* and *H*, denoted by $G \Box H$, is a graph with a vertex set $V(G \Box H)=V(G)\times V(H)$ and $(u_{1}, v_{1})(u_{2}, v_{2}) \in E(G \Box H)$ whenever [$u_{1} = u_{2}$ and $v_{1}v_{2} \in E(H)$] or [$u_{1}u_{2} \in E(G)$ and $v_{1} = v_{2}$].

Now we state distinct properties of the Cartesian product of graphs in form of the following lemma.

### Lemma 1

*Let* $G_{1}$ *and* $G_{2}$ *be graphs of orders* $n_{1}$, $n_{2}$ *and sizes* $m_{1}$, $m_{2}$, *respectively*. *Then we have*:

- $\vert V(G_{1}\Box G_{2})\vert = \vert V(G_{1})\vert \vert V(G_{2})\vert $ *and* $\vert E(G_{1}\Box G_{2})\vert = \vert V(G_{2})\vert \vert E(G_{1})\vert + \vert V(G_{1})\vert \vert E(G_{2})\vert $,
- $\operatorname{deg}_{G_{1}\Box G_{2}}(u,v)=\operatorname{deg}_{G_{1}}(u)+ \operatorname{deg}_{G_{2}}(v) $.

For a connected graph *G*, define four related graphs $S(G)$, $R(G)$, $Q(G)$ and $T(G)$ as follows:

1. $S(G)$ is the graph obtained by inserting an additional vertex in each edge of *G*, i.e., replacing each edge of *G* by a path of length 2. The graph $S(G)$ is also known as a subdivision graph of *G*.
2. $R(G)$ is the graph obtained by adding a new vertex corresponding to each edge of *G*, then joining each new vertex to the end vertices of the corresponding edge.
3. $Q(G)$ is the graph obtained by inserting a new vertex into each edge of *G*, then joining with edges those pairs of new vertices on adjacent edges of *G*.
4. $T(G)$ has as its vertices, the edges and vertices of *G*. Adjacency in $T(G)$ is defined as adjacency or incidence for the corresponding elements of *G*. The graph $T(G)$ is called the total graph of *G*.

**Figure 1:**
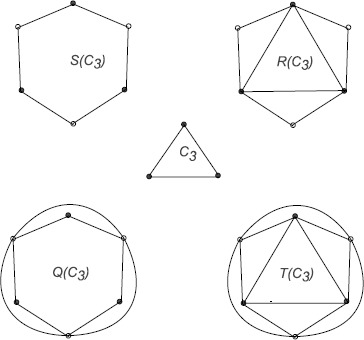
**The graphs** ***G*** **,** $\pmb{S(G)}$ **,** $\pmb{R(G)}$ **,** $\pmb{Q(G)}$ **and** $\pmb{T(G)}$ **.**

The four operations, $S(G)$, $R(G)$, $Q(G)$ and $T(G)$ on a graph *G* are illustrated in Figure 1.

Eliasi and Taeri [20] introduced four new operations that are based on $S(G)$, $R(G)$, $Q(G)$, $T(G)$ as follows.

**Figure 2:**
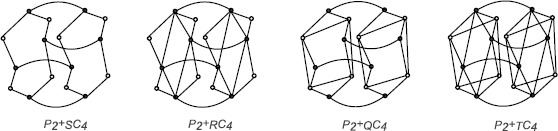
**The graphs** $\pmb{P_{2}+_{S}C_{4}}$ **,** $\pmb{P_{2}+_{R}C_{4}}$ **,** $\pmb{P_{2}+_{Q}C_{4}}$ **and** $\pmb{P_{2}+_{T}C_{4}}$ **.**

Let *F* be one of the symbols *S*, *R*, *Q* or *T*. The *F*-sum, denoted by $G+_{F}H$, of graphs *G* and *H* having orders $n_{1}$ and $n_{2}$, respectively, is a graph with the set of vertices $V(G+_{F}H)=(V(G)\cup E(G))\times V(H)$ and $(u_{1}, v_{1})(u_{2}, v_{2}) \in E(G +_{F}H)$ if and only if [$u_{1} = u_{2} \in V(G)$ and $v_{1}v_{2} \in E(H)$] or [$v_{1} = v_{2} \in V(H)$ and $u_{1}u_{2} \in E(F(G))$], where $F\in \{S, R, Q, T\}$. $G +_{F}H$ consists of $n_{2}$ copies of the graph $F(G)$, and we label these copies by vertices of *H*. The vertices in each copy have two types, the vertices in $V(G)$ (black vertices) and the vertices in $E(G)$ (white vertices). Now we join only black vertices with the same name in $F(G)$ in which their corresponding labels are adjacent in *H*. The graphs $P_{2}+_{F}C _{4}$ are shown in Figure 2.

Eliasi and Taeri [20] computed the expression for the Wiener index of four graph operations which are based on these graphs $S(G)$, $R(G)$, $Q(G)$, and $T(G)$ in terms of $W(F(G))$ and $W(H)$. Deng et al. [21] computed the first and second Zagreb indices for the graph operations $S(G)$, $R(G)$, $Q(G)$ and $T(G)$. Akhter and Imran computed bounds for the general sum-connectivity index of *F*-sums of graphs [22]. Some explicit computing formulas for different topological indices of some important graphs can be found in [7, 23–27].

To avoid computational complications, it is important to express the formulas for the product of *F*-sum of graphs in terms of their factor graphs. So, we presented bounds for the first Zagreb index, the ABC-index, the third Zagreb index, the augmented Zagreb index, the *F*-index, the first multiple Zagreb index and the GA-index for the Cartesian product of *F*-sum of graphs in form of its factor graphs.

## Main results and discussions

This section is meant for determination of bounds for the first Zagreb, the third Zagreb, the augmented Zagreb, the first multiple Zagreb, ABC and GA indices of the Cartesian product of *F*-sum of graphs in terms of their factor graphs. Bounds for the *F*-index and the Narumi-Katayama index are also discussed. The following lemmas are useful for determination of these bounds.

In the following lemma, we compute the size of *F*-sum of graphs for $F=S$.

### Lemma 2

*If* $G=G_{1}+_{S}G_{2}$, *then the size of* *G* *is* $n_{1} m_{2}+2n_{2} m_{1}$, *where* $\vert V(G_{1})\vert =n_{1}$, $\vert V(G_{2})\vert =n_{2}$, $\vert E(G_{1})\vert =m_{1}$ *and* $\vert E(G_{2})\vert =m_{2}$.

### Proof

We know that $S(G_{1})$ is a subdivision of $G_{1}$, therefore the size of $S(G_{1})$ is $2\vert E(G_{1})\vert =2m_{1}$.

Hence $\vert E(G)\vert =\vert V(G_{1})\vert \vert E(G_{2})\vert +2\vert V(G_{2})\vert \vert E(G_{1})\vert =n_{1} m_{2}+2n_{2} m_{1}$. □

In the following lemma, we compute the size of *F*-sum of graphs for $F=Q$.

### Lemma 3

*If* $G=G_{1}+_{Q}G_{2}$, *then the size of* *G* *is* $n_{1} m_{2}+\frac{n_{2} m_{1}(m_{1}+3)}{2}$, *where* $\vert V(G_{1})\vert =n_{1}$, $\vert V(G_{2})\vert =n_{2}$, $\vert E(G_{1})\vert =m_{1}$ *and* $\vert E(G_{2})\vert =m_{2}$.

### Proof

By using combinations, the size of $Q(G_{1})=2m_{1}+ ^{m_{1}}C_{2}=\frac{m_{1}(m_{1}+3)}{2}$.

Hence $\vert E(G)\vert =\vert V(G_{1})\vert \vert E(G_{2})\vert +\vert V(G_{2})\vert \vert E[Q(G_{1})]\vert =n_{1} m_{2}+\frac{n_{2}m_{1}(m_{1}+3)}{2}$. □

In the following lemma, we compute the size of *F*-sum of graphs for $F=R$.

### Lemma 4

*If* $G=G_{1}+_{R}G_{2}$, *then the size of* *G* *is* $n_{1} m_{2}+3n_{2} m_{1}$, *where* $\vert V(G_{1})\vert =n_{1}$, $\vert V(G_{2})\vert =n_{2}$, $\vert E(G_{1})\vert =m_{1}$ *and* $\vert E(G_{2})\vert =m_{2}$.

### Proof

We know that the size of $R(G_{1})$ is equal to three times the size of $G_{1}$, therefore the size of $R(G_{1})$ is $3\vert E(G_{1})\vert =3m_{1}$.

Hence $\vert E(G)\vert =\vert V(G_{1})\vert \vert E(G_{2})\vert +3\vert V(G_{2})\vert \vert E(G_{1})\vert =n_{1} m_{2}+3n_{2} m_{1}$. □

In the following lemma, we compute the size of *F*-sum of graphs for $F=T$.

### Lemma 5

*If* $G=G_{1}+_{T}G_{2}$, *then the size of* *G* *is* $n_{1} m_{2}+\frac{n_{2} m_{1}(m_{1}+5)}{2}$, *where* $\vert V(G_{1})\vert =n_{1}$, $\vert V(G_{2})\vert =n_{2}$, $\vert E(G_{1})\vert =m_{1}$ *and* $\vert E(G_{2})\vert =m_{2}$.

### Proof

By using combinations, the size of $T(G_{1})=3m_{1}+ ^{m_{1}}C_{2}=\frac{m_{1}(m_{1}+5)}{2}$.

Hence $\vert E(G)\vert =\vert V(G_{1})\vert \vert E(G_{2})\vert +\vert V(G_{2})\vert \vert E[T(G_{1})]\vert =n_{1} m_{2}+\frac{n_{2}m_{1}(m_{1}+5)}{2}$. □

Let $G_{1}$, $G_{2}$, $H_{1}$, $H_{2}$ be simple, connected graphs such that $\vert V(G_{1})\vert =n_{1}$, $\vert V(G_{2})\vert =n_{2}$, $\vert V(H_{1})\vert = {n'_{1}}$, $\vert V(H_{2})\vert ={n'_{2}}$, $\vert E(G_{1})\vert =m_{1}$, $\vert E(G_{2})\vert =m_{2}$, $\vert E(H_{1})\vert = {m'_{1}}$ and $\vert E(H_{2})\vert ={m'_{2}}$.

In the following theorem the lower and upper bounds for the first Zagreb, the third Zagreb, the atom-bond connectivity (ABC), the augmented Zagreb, the first multiple Zagreb and geometric-arithmetic (GA) indices of the Cartesian product of *F*-sum of graphs in terms of their factor graphs for $F=S$ are determined.

### Theorem 1

*Let* $G=G_{1}+_{S}H_{1}$ *and* $H=G_{2}+_{S}H_{2}$, *then*

- $2\alpha (\delta_{G}+\delta_{H} )\leq M_{1}(G\Box H) \leq 2\alpha (\Delta_{G}+\Delta_{H} )$,
- $\alpha \frac{\sqrt{2(\delta _{G}+\delta _{H}-1)}}{ \Delta _{G}+\Delta _{H}}\leq \operatorname{ABC}(G\Box H)\leq \alpha \frac{\sqrt{2(\Delta _{G}+\Delta _{H}-1)}}{\delta _{G}+\delta _{H}}$,
- $4\alpha (\delta_{G}+\delta_{H} )^{2}\leq M_{3}(G\Box H) \leq 4\alpha (\Delta_{G}+\Delta_{H} )^{2}$,
- $\frac{1}{8}\alpha [\frac{(\delta_{G}+\delta_{H})^{2}}{\Delta _{G}+\Delta_{H}-1} ]^{3}\leq \operatorname{AZI}(G\Box H)\leq \frac{1}{8}\alpha [ \frac{ (\Delta_{G}+\Delta_{H} )^{2}}{\delta_{G}+\delta_{H}-1} ]^{3}$,
- $2^{\alpha } (\delta_{G}+\delta_{H} )^{\alpha }\leq \operatorname{PM}_{1}(G \Box H) \leq 2^{\alpha } (\Delta_{G}+\Delta_{H} )^{\alpha }$,
- $\alpha (\frac{\delta _{G}+\delta _{H}}{\Delta _{G}+\Delta _{H}} ) \leq \operatorname{GA}(G\Box H) \leq \alpha (\frac{\Delta _{G}+\Delta _{H}}{\delta _{G}+\delta _{H}} )$,

*where* $\alpha =n_{1}(n'_{1}+m'_{1})(m_{2}n'_{2}+2n_{2}m'_{2}) +n_{2}( {n}'_{2}+m'_{2})(m_{1}n'_{1}+2n_{1}m'_{1})$, $\delta_{G}+\delta_{H}=\delta_{G_{1}}+\delta_{G_{2}}+\delta_{H_{1}}+ \delta_{H_{2}}$ *and* $\Delta_{G}+\Delta_{H}=\Delta_{G_{1}}+\Delta_{G _{2}}+\Delta_{H_{1}}+\Delta_{H_{2}}$.

### Proof

Let *G* and *H* be the graphs with vertex sets $\{u_{1},u_{2},\ldots,u _{n_{1}(n'_{1}+m'_{1})}\}$ and $\{v_{1},v_{2},\ldots, v_{n_{2}(n'_{2}+m'_{2})} \}$, respectively. Then

(a) By definition,

1 $$\begin{aligned}& \begin{aligned}&M_{1}(G\Box H)= \sum_{(u_{i},v_{j})(u_{k},v_{l}) \in E(G\Box H)} \bigl[\operatorname{deg}_{G\Box H}(u _{i},v_{j})+\operatorname{deg}_{G\Box H}(u_{k},v_{l}) \bigr] \\ &\hphantom{M_{1}(G\Box H)}= \sum_{(u_{i},v_{j})(u_{k},v_{l}) \in E(G\Box H), i\neq k} \bigl[\operatorname{deg}_{G\Box H}(u_{i},v_{j})+\operatorname{deg}_{G\Box H}(u_{k},v_{l}) \bigr] \\ &\hphantom{M_{1}(G\Box H)=}{}+ \sum_{(u_{i},v_{j})(u_{k},v_{l}) \in E(G\Box H), j\neq l} \bigl[\operatorname{deg}_{G\Box H}(u_{i},v_{j})+\operatorname{deg}_{G\Box H}(u_{k},v_{l}) \bigr], \\ & M_{1}(G\Box H)= \sum_{u_{i} \in V(G)} \sum_{v_{j}v_{l} \in E(H)} \bigl[\operatorname{deg}_{G\Box H}(u_{i},v_{j})+ \operatorname{deg}_{G \Box H}(u_{i},v_{l}) \bigr] \\ &\hphantom{M_{1}(G\Box H)=}{}+ \sum_{v_{j} \in V(H)} \sum _{u_{i}u_{k} \in E(G)} \bigl[\operatorname{deg}_{G\Box H}(u_{i},v_{j})+ \operatorname{deg}_{G \Box H}(u_{k},v_{j}) \bigr]. \end{aligned} \end{aligned}$$

By using Lemma 1, part (b), we obtain

$$\operatorname{deg}_{G\Box H}(u_{i},v_{j})+ \operatorname{deg}_{G\Box H} (u_{k},v_{l})=\operatorname{deg}_{G}(u_{i})+\operatorname{deg}_{H}(v_{j})+ \operatorname{deg}_{G}(u_{k})+ \operatorname{deg}_{H}(v_{l}). $$

Since, for any vertex $u\in V(G)$, $\operatorname{deg}_{G}(u)\leq \Delta_{G}$ and $\operatorname{deg}_{G}(u)\geq \delta_{G}$, therefore, by using these facts, we obtain

$$\begin{aligned}& \operatorname{deg}_{G\Box H}(u_{i},v_{j})+\operatorname{deg}_{G\Box H}(u_{k},v_{l}) \leq \Delta _{G}+ \Delta_{H}+\Delta_{G}+\Delta_{H}, \end{aligned}$$

which implies the inequality

2 $$ \operatorname{deg}_{G\Box H}(u_{i},v_{j})+\operatorname{deg}_{G\Box H}(u_{k},v_{l}) \leq 2(\Delta _{G}+ \Delta_{H}). $$

By using inequality (2) in equation (1), we obtain

$$\begin{aligned} M_{1}(G\Box H) =& \sum_{(u_{i},v_{j})(u_{k},v_{l}) \in E(G\Box H), i\neq k} \bigl[\operatorname{deg}_{G\Box H}(u_{i},v_{j})+\operatorname{deg}_{G\Box H}(u_{k},v_{l}) \bigr] \\ &{}+ \sum_{(u_{i},v_{j})(u_{k},v_{l}) \in E(G\Box H), j\neq l} \bigl[\operatorname{deg}_{G\Box H}(u_{i},v_{j})+ \operatorname{deg}_{G\Box H}(u_{k},v_{l}) \bigr] \\ =& \sum_{u_{i} \in V(G)} \sum_{v_{j}v_{l} \in E(H)} \bigl[\operatorname{deg}_{G\Box H}(u_{i},v_{j})+\operatorname{deg}_{G \Box H}(u_{i},v_{l}) \bigr] \\ &{}+ \sum_{v_{j} \in V(H)} \sum_{u_{i}u_{k} \in E(G)} \bigl[\operatorname{deg}_{G\Box H}(u_{i},v_{j})+\operatorname{deg}_{G \Box H}(u_{k},v_{j}) \bigr] \\ \leq& \bigl\vert V(G)\bigr\vert \bigl\vert E(H)\bigr\vert 2(\Delta _{G}+\Delta_{H})+\bigl\vert E(G)\bigr\vert \bigl\vert V(H)\bigr\vert 2(\Delta_{G}+ \Delta_{H}). \end{aligned}$$

Since $\vert V(G)\vert =n_{1}(n'_{1}+m'_{1})$, $\vert V(H)\vert =n_{2}(n'_{2}+m'_{2})$, $\vert E(G)\vert =m_{1}n'_{1}+2n_{1}m'_{1}$, $\vert E(H)\vert =m_{2}n'_{2}+2n _{2}m'_{2}$, $\Delta_{G}=\Delta_{G_{1}}+\Delta_{H_{1}}$ and $\Delta_{H}=\Delta_{G_{2}}+\Delta_{H_{2}}$, therefore we obtain

3 $$\begin{aligned} M_{1}(G\Box H) \leq &2 \bigl[n_{1} \bigl(n'_{1}+m'_{1} \bigr) \bigl(m_{2}n'_{2}+2n_{2}m'_{2} \bigr) +n_{2} \bigl(n'_{2}+m'_{2} \bigr) \bigl(m _{1}n'_{1}+2n_{1}m'_{1} \bigr) \bigr] \\ &{}\times (\Delta_{G_{1}}+\Delta_{H_{1}}+ \Delta_{G_{2}}+ \Delta_{H_{2}} ). \end{aligned}$$

By using similar arguments with $\operatorname{deg}_{G}(u)\geq \delta_{G}$, we obtain

4 $$\begin{aligned} M_{1}(G\Box H) \geq& 2 \bigl[n_{1} \bigl(n'_{1}+m'_{1} \bigr) \bigl(m_{2}n'_{2}+2n_{2}m'_{2} \bigr) +n_{2} \bigl(n'_{2}+m'_{2} \bigr) \bigl(m _{1}n'_{1}+2n_{1}m'_{1} \bigr) \bigr] \\ &{}\times (\delta_{G_{1}}+\delta_{H_{1}}+ \delta_{G_{2}}+ \delta_{H_{2}} ). \end{aligned}$$

Hence part (a) of the theorem is proved by substituting $n_{1}(n'_{1}+m'_{1})(m_{2}n'_{2}+2n_{2}m'_{2})+n_{2}(n'_{2}+m'_{2})(m_{1}n'_{1}+2n_{1}m'_{1})=\alpha $ in inequalities (3) and (4).

(b) By definition,

5 $$\begin{aligned}& \begin{aligned}&\operatorname{ABC}(G\Box H)= \sum_{(u_{i},v_{j})(u_{k},v_{l}) \in E(G\Box H)} \sqrt{ \frac{\operatorname{deg}_{G\Box H}(u_{i},v_{j})+ \operatorname{deg}_{G\Box H}(u_{k},v_{l})-2}{\operatorname{deg}_{G\Box H}(u _{i},v_{j}) \operatorname{deg}_{G\Box H}(u_{k},v_{l})}}. \\ & \operatorname{ABC}(G\Box H)= \sum_{u_{i} \in V(G)} \sum _{v_{j}v_{l} \in E(H)}\sqrt{ \frac{\operatorname{deg}_{G\Box H}(u_{i},v_{j})+ \operatorname{deg}_{G\Box H}(u_{i},v_{l})-2}{\operatorname{deg}_{G\Box H}(u_{i},v_{j}) \operatorname{deg}_{G\Box H}(u _{i},v_{l})}} \\ &\hphantom{\operatorname{ABC}(G\Box H)=}{}+ \sum_{v_{j} \in V(H)} \sum _{u_{i}u_{k} \in E(G)} \sqrt{\frac{\operatorname{deg}_{G\Box H}(u_{i},v_{j})+ \operatorname{deg}_{G\Box H}(u_{k},v_{j})-2}{\operatorname{deg}_{G\Box H}(u_{i},v_{j}) \operatorname{deg}_{G\Box H}(u _{k},v_{j})}}. \end{aligned} \end{aligned}$$

By using Lemma 1, part (b), we obtain

$$\operatorname{deg}_{G\Box H}(u_{i},v_{j}) \operatorname{deg}_{G\Box H}(u_{k},v_{l})= \bigl[\operatorname{deg}_{G}(u _{i})+ \operatorname{deg}_{H}(v_{j}) \bigr] \bigl[\operatorname{deg}_{G}(u_{k})+ \operatorname{deg}_{H}(v_{l}) \bigr]. $$

Since, for any vertex $u\in V(G)$, $\operatorname{deg}_{G}(u)\leq \Delta_{G}$ and $\operatorname{deg}_{G}(u)\geq \delta_{G}$, therefore, by using these facts, we obtain

$$\operatorname{deg}_{G\Box H}(u_{i},v_{j}) \operatorname{deg}_{G\Box H}(u_{k},v_{l}) \leq (\Delta _{G} + \Delta_{H}) (\Delta_{G} +\Delta_{H}), $$

which implies the inequality

6 $$ \operatorname{deg}_{G\Box H}(u_{i},v_{j}) \operatorname{deg}_{G\Box H}(u_{k},v_{l}) \leq ( \Delta_{G} + \Delta_{H} )^{2}. $$

By using inequalities (2) and (6) in equation (5), we obtain

$$\begin{aligned} \operatorname{ABC}(G\Box H) =& \sum_{(u_{i},v_{j})(u_{k},v_{l}) \in E(G\Box H), i\neq k} \sqrt{ \frac{\operatorname{deg}_{G\Box H}(u_{i},v_{j})+ \operatorname{deg}_{G\Box H}(u_{k},v_{l})-2}{\operatorname{deg}_{G\Box H}(u _{i},v_{j}) \operatorname{deg}_{G\Box H}(u_{k},v_{l})}} \\ &{}+ \sum_{(u_{i},v_{j})(u_{k},v_{l}) \in E(G\Box H), j\neq l} \sqrt{\frac{\operatorname{deg}_{G\Box H}(u_{i},v_{j})+ \operatorname{deg}_{G\Box H}(u_{k},v_{l})-2}{\operatorname{deg}_{G\Box H}(u _{i},v_{j}) \operatorname{deg}_{G\Box H}(u_{k},v_{l})}} \\ =& \sum_{u_{i} \in V(G)} \sum_{v_{j}v_{l} \in E(H)} \sqrt{\frac{\operatorname{deg}_{G\Box H}(u_{i},v_{j})+ \operatorname{deg}_{G\Box H}(u_{i},v_{l})-2}{\operatorname{deg}_{G\Box H}(u_{i},v_{j}) \operatorname{deg}_{G\Box H}(u _{i},v_{l})}} \\ &{}+ \sum_{v_{j} \in V(H)} \sum_{u_{i}u_{k} \in E(G)} \sqrt{\frac{\operatorname{deg}_{G\Box H}(u_{i},v_{j})+ \operatorname{deg}_{G\Box H}(u_{i},v_{l})-2}{\operatorname{deg}_{G\Box H}(u_{i},v_{j}) \operatorname{deg}_{G\Box H}(u _{i},v_{l})}} \\ &{}\leq \bigl\vert V(G)\bigr\vert \bigl\vert E(H)\bigr\vert \sqrt{ \frac{2( \Delta_{G}+\Delta_{H})-2}{(\Delta_{G}+\Delta_{H})^{2}}}+\bigl\vert E(G)\bigr\vert \bigl\vert V(H)\bigr\vert \sqrt{\frac{2( \Delta_{G}+\Delta_{H})-2}{(\Delta_{G}+\Delta_{H})^{2}}}. \end{aligned}$$

Since $\vert V(G)\vert =n_{1}(n'_{1}+m'_{1})$, $\vert V(H)\vert =n_{2}(n'_{2}+m'_{2})$, $\vert E(G)\vert =m_{1}n'_{1}+2n_{1}m'_{1}$, $\vert E(H)\vert =m_{2}n'_{2}+2n_{2}m'_{2}$, $\Delta_{G}=\Delta_{G_{1}}+\Delta_{H_{1}}$ and $\Delta_{H}=\Delta_{G_{2}}+\Delta_{H_{2}}$, therefore we get

7 $$\begin{aligned} \operatorname{ABC}(G\Box H) \leq & \bigl[n_{1} \bigl(n'_{1}+m'_{1} \bigr) \bigl(m_{2}n'_{2}+2n_{2}m'_{2} \bigr) +n_{2} \bigl(n'_{2}+m'_{2} \bigr) \bigl(m_{1}n'_{1}+2n_{1}m'_{1} \bigr) \bigr] \\ &{}\times \frac{\sqrt{2( \Delta_{G_{1}}+\Delta_{G_{2}}+\Delta_{H_{1}}+\Delta_{H_{2}}-1)}}{ \delta_{G_{1}}+\delta_{G_{2}} +\delta_{H_{1}}+\delta_{H_{2}}}. \end{aligned}$$

By using similar arguments with $\operatorname{deg}_{G}(u)\geq \delta_{G}$, we obtain

8 $$\begin{aligned} \operatorname{ABC}(G\Box H) \geq & \bigl[n_{1} \bigl(n'_{1}+m'_{1} \bigr) \bigl(m_{2}n'_{2}+2n_{2}m'_{2} \bigr) +n_{2} \bigl(n'_{2}+m'_{2} \bigr) \bigl(m_{1}n'_{1}+2n_{1}m'_{1} \bigr) \bigr] \\ &{}\times \frac{\sqrt{2( \delta_{G_{1}}+\delta_{G_{2}}+\delta_{H_{1}}+\delta_{H_{2}}-1)}}{ \Delta_{G_{1}}+\Delta_{G_{2}} +\Delta_{H_{1}}+\Delta_{H_{2}}}. \end{aligned}$$

Hence from inequalities (7) and (8), part (b) of the theorem is proved.

(c) By definition,

9 $$\begin{aligned}& \begin{aligned}&M_{3}(G\Box H)= \sum_{(u_{i},v_{j})(u_{k},v_{l}) \in E(G\Box H)} \bigl[\operatorname{deg}_{G\Box H}(u _{i},v_{j})+\operatorname{deg}_{G\Box H}(u_{k},v_{l}) \bigr]^{2}, \\ & M_{3}(G\Box H)= \sum_{u_{i} \in V(G)} \sum_{v_{j}v_{l} \in E(H)} \bigl[\operatorname{deg}_{G\Box H}(u_{i},v_{j})+ \operatorname{deg}_{G \Box H}(u_{i},v_{l}) \bigr]^{2} \\ &\hphantom{ M_{3}(G\Box H)=}{}+ \sum_{v_{j} \in V(H)} \sum _{u_{i}u_{k} \in E(G)} \bigl[\operatorname{deg}_{G\Box H}(u_{i},v_{j})+ \operatorname{deg}_{G \Box H}(u_{k},v_{j}) \bigr]^{2}. \end{aligned} \end{aligned}$$

By using inequality (2) in equation (9) and adopting the same procedure as in part (a) of this theorem,

10 $$\begin{aligned} M_{3}(G\Box H) \leq & 4 \bigl[n_{1} \bigl(n'_{1}+m'_{1} \bigr) \bigl(m_{2}n'_{2}+2n_{2}m'_{2} \bigr) +n_{2} \bigl(n'_{2}+m'_{2} \bigr) \bigl(m _{1}n'_{1}+2n_{1}m'_{1} \bigr) \bigr] \\ &{}\times (\Delta_{G_{1}}+\Delta_{H_{1}}+ \Delta_{G_{2}}+ \Delta_{H_{2}} )^{2} \end{aligned}$$

and

11 $$\begin{aligned} M_{3}(G\Box H) \geq &4 \bigl[n_{1} \bigl(n'_{1}+m'_{1} \bigr) \bigl(m_{2}n'_{2}+2n_{2}m'_{2} \bigr) +n_{2} \bigl(n'_{2}+m'_{2} \bigr) \bigl(m _{1}n'_{1}+2n_{1}m'_{1} \bigr) \bigr] \\ &{}\times (\delta_{G_{1}}+\delta_{H_{1}}+ \delta_{G_{2}}+ \delta_{H_{2}} )^{2}. \end{aligned}$$

Inequalities (10) and (11) complete the proof of part (c) of the theorem.

(d) By definition,

12 $$\begin{aligned}& \begin{aligned}&\operatorname{AZI}(G\Box H)= \sum_{(u_{i},v_{j})(u_{k},v_{l}) \in E(G\Box H)} \biggl[ \frac{\operatorname{deg}_{G \Box H}(u_{i},v_{j}).\operatorname{deg}_{G\Box H}(u_{k},v_{l})}{\operatorname{deg}_{G\Box H}(u_{i},v _{j})+\operatorname{deg}_{G\Box H}(u_{k},v_{l})-2} \biggr]^{3}. \\ & \operatorname{AZI}(G\Box H)= \sum_{u_{i} \in V(G)} \sum _{v_{j}v_{l} \in E(H)} \biggl[ \frac{\operatorname{deg}_{G\Box H}(u_{i},v_{j}) \operatorname{deg}_{G\Box H}(u_{k},v_{l})}{\operatorname{deg}_{G\Box H}(u_{i},v_{j})+\operatorname{deg}_{G\Box H}(u _{k},v_{l})-2} \biggr]^{3} \\ &\hphantom{\operatorname{AZI}(G\Box H)=}{}+ \sum_{v_{j} \in V(H)} \sum _{u_{i}u_{k} \in E(G)} \biggl[\frac{\operatorname{deg}_{G\Box H}(u_{i},v_{j}) \operatorname{deg}_{G\Box H}(u_{k},v_{l})}{\operatorname{deg}_{G\Box H}(u_{i},v_{j})+\operatorname{deg}_{G\Box H}(u _{k},v_{l})-2} \biggr]^{3}. \end{aligned} \end{aligned}$$

By using inequalities (2) and (6) in equation (12) and adopting the same procedure as in parts (a) and (b) of this theorem, we obtain

13 $$\begin{aligned} \operatorname{AZI}(G\Box H) \leq &\frac{1}{8} \bigl[n_{1} \bigl(n'_{1}+m'_{1} \bigr) \bigl(m_{2}n'_{2}+2n _{2}m'_{2} \bigr) +n_{2} \bigl(n'_{2}+m'_{2} \bigr) \bigl(m_{1}n'_{1}+2n_{1}m'_{1} \bigr) \bigr] \\ &{}\times \biggl[\frac{(\Delta_{G_{1}}+\Delta_{H_{1}}+\Delta_{G_{2}}+\Delta_{H _{2}})^{2}}{\delta_{G_{1}}+\delta_{H_{1}} +\delta_{G_{2}}+\delta_{H _{2}}-1} \biggr]^{3} \end{aligned}$$

and

14 $$\begin{aligned} \operatorname{AZI}(G\Box H) \geq &\frac{1}{8} \bigl[n_{1} \bigl(n'_{1}+m'_{1} \bigr) \bigl(m_{2}n'_{2}+2n _{2}m'_{2} \bigr) +n_{2} \bigl(n'_{2}+m'_{2} \bigr) \bigl(m_{1}n'_{1}+2n_{1}m'_{1} \bigr) \bigr] \\ &{}\times \biggl[\frac{(\delta_{G_{1}}+\delta_{H_{1}}+\delta_{G_{2}}+\delta_{H _{2}})^{2}}{\Delta_{G_{1}}+\Delta_{H_{1}} +\Delta_{G_{2}}+\Delta_{H _{2}}-1} \biggr]^{3}. \end{aligned}$$

Inequalities (13) and (14) complete the proof of part (d) of the theorem.

(e) By definition,

15 $$ \operatorname{PM}_{1}(G\Box H)= \prod_{(u_{i},v_{j})(u_{k},v_{l}) \in E(G\Box H)} \bigl[\operatorname{deg}_{G\Box H}(u _{i},v_{j})+\operatorname{deg}_{G\Box H}(u_{k},v_{l}) \bigr]. $$

By using inequality (2) in equation (15) and adopting the same procedure as in part (i) of this theorem,

16 $$ \operatorname{PM}_{1}(G\Box H)\leq \bigl[2(\Delta_{G}+ \Delta_{H}) \bigr]^{n_{1}(n'_{1}+m'_{1})(m _{2}n'_{2}+2n_{2}m'_{2}) +n_{2}(n'_{2}+m'_{2})(m_{1}n'_{1}+2n_{1}m'_{1})} $$

and

17 $$ \operatorname{PM}_{1}(G\Box H)\geq \bigl[2(\delta_{G}+ \delta_{H}) \bigr]^{n_{1}(n'_{1}+m'_{1})(m _{2}n'_{2}+2n_{2}m'_{2}) +n_{2}(n'_{2}+m'_{2})(m_{1}n'_{1}+2n_{1}m'_{1})}. $$

Hence from inequalities (16) and (17), part (e) of the theorem is proved.

(f) By definition,

18 $$ \operatorname{GA}(G\Box H)= \sum_{(u_{i},v_{j})(u_{k},v_{l}) \in E(G\Box H)}{ \frac{2 \sqrt{\operatorname{deg}_{G\Box H}(u_{i},v_{j}) \operatorname{deg}_{G\Box H}(u_{k},v_{l})}}{\operatorname{deg}_{G\Box H}(u _{i},v_{j})+ \operatorname{deg}_{G\Box H}(u_{k},v_{l})}}. $$

By using inequalities (2) and (6) in equation (18) and adopting the same procedure as in part (b) of this theorem,

19 $$\begin{aligned} \operatorname{GA}(G\Box H) \leq & \bigl[n_{1} \bigl(n'_{1}+m'_{1} \bigr) \bigl(m_{2}n'_{2}+2n_{2}m'_{2} \bigr) +n_{2} \bigl(n'_{2}+m'_{2} \bigr) \bigl(m_{1}n'_{1}+2n_{1}m'_{1} \bigr) \bigr] \\ &{}\times \biggl(\frac{ \Delta_{G_{1}}+\Delta_{G_{2}}+\Delta_{H_{1}}+\Delta_{H_{2}}}{ \delta_{G_{1}}+\delta_{G_{2}}+\delta_{H_{1}} +\delta_{H_{2}}} \biggr) \end{aligned}$$

and

20 $$\begin{aligned} \operatorname{GA}(G\Box H) \geq & \bigl[n_{1} \bigl(n'_{1}+m'_{1} \bigr) \bigl(m_{2}n'_{2}+2n_{2}m'_{2} \bigr) +n_{2} \bigl(n'_{2}+m'_{2} \bigr) \bigl(m_{1}n'_{1}+2n_{1}m'_{1} \bigr) \bigr] \\ &{}\times \biggl(\frac{ \delta_{G_{1}}+\delta_{G_{2}}+\delta_{H_{1}}+\delta_{H_{2}}}{ \Delta_{G_{1}}+\Delta_{G_{2}}+\Delta_{H_{1}} +\Delta_{H_{2}}} \biggr). \end{aligned}$$

Hence from inequalities (19) and (20), part (f) of the theorem is proved. □

We determine the lower and upper bounds for the *F*-index and the Narumi-Katayama index of the Cartesian product of *F*-sum of graphs in terms of their factor graphs for $F=S$.

### Corollary 1

*Let* $G=G_{1}+_{S}H_{1}$ *and* $H=G_{2}+_{S}H_{2}$, *then*

- $2\alpha (\delta_{G}+\delta_{H} )^{2}\leq F(G\Box H) \leq 2\alpha (\Delta_{G}+\Delta_{H} )^{2}$,
- $[2(\delta_{G}+\delta_{H}) ]^{\frac{\alpha }{2}}\leq \operatorname{NK}(G \Box H) \leq [2(\Delta_{G}+\Delta_{H}) ]^{\frac{\alpha }{2}}$,

*where* $\alpha =n_{1}(n'_{1}+m'_{1})(m_{2}n'_{2}+2n_{2}m'_{2})+n_{2}(n'_{2}+m'_{2})(m _{1}n'_{1}+2n_{1}m'_{1})$, $\delta_{G}+\delta_{H}=\delta_{G_{1}}+ \delta_{G_{2}}+\delta_{H_{1}}+\delta_{H_{2}}$ *and* $\Delta_{G}+\Delta _{H}=\Delta_{G_{1}}+\Delta_{G_{2}}+\Delta_{H_{1}}+\Delta_{H_{2}}$.

### Proof

(a) By using the relation $F(G)=M_{3}(G)-2 M_{2}(G)$, in Theorem 1, we obtain the required result.

(b) By using the relation $\operatorname{NK}(G)=\sqrt{\operatorname{PM}_{1}(G)}$, in Theorem 1, we obtain the required result. □

In the following theorem the lower and upper bounds for the first Zagreb, the third Zagreb, the atom-bond connectivity (ABC), the augmented Zagreb, the first multiple Zagreb and geometric-arithmetic (GA) indices of the Cartesian product of *F*-sum of graphs in terms of their factor graphs for $F=Q$ are determined.

### Theorem 2

*Let* $G=G_{1}+_{Q}H_{1}$ *and* $H=G_{2}+_{Q}H_{2}$, *then*

- $2\beta (\delta _{G}+\delta _{H} )\leq M_{1}(G\Box H)\leq 2\beta (\Delta _{G}+\Delta _{H} )$,
- $\beta \frac{\sqrt{2(\delta _{G}+\delta _{H}-1)}}{\Delta _{G}+\Delta _{H}}\leq \operatorname{ABC}(G\Box H)\leq \beta \frac{\sqrt{2(\Delta _{G}+\Delta _{H}-1)}}{\delta _{G}+\delta _{H}}$,
- $4\beta (\delta _{G}+\delta _{H} )^{2}\leq M_{3}(G\Box H)\leq 4\beta (\Delta _{G}+\Delta _{H} )^{2}$,
- $\frac{1}{8}\beta [\frac{(\delta _{G}+\delta _{H})^{2}}{\Delta _{G}+\Delta _{H}-1} ]^{3}\leq \operatorname{AZI}(G\Box H)\leq \frac{1}{8}\beta [\frac{ (\Delta _{G}+\Delta _{H} )^{2}}{\delta _{G}+\delta _{H}-1} ]^{3}$,
- $2^{\beta } (\delta _{G}+\delta _{H} )^{\beta }\leq \operatorname{PM}_{1}(G\Box H) \leq 2^{\beta } (\Delta _{G}+\Delta _{H} )^{\beta }$,
- $\beta (\frac{\delta _{G}+\delta _{H}}{\Delta _{G}+\Delta _{H}} )\leq \operatorname{GA}(G\Box H) \leq \beta (\frac{\Delta _{G}+\Delta _{H}}{\delta _{G}+\delta _{H}} )$,

*where* $\beta =\frac{1}{2} [n_{1}(n'_{1}+m'_{1})\{2n_{2} m'_{2}+n'_{2} m_{2}(m_{2}+3)\} +n_{2}({n}'_{2}+m'_{2})\{2n_{1} m'_{1}+n'_{1} m_{1}(m_{1}+3)\} ]$, $\delta _{G}+\delta _{H}=\delta _{G_{1}}+\delta _{G_{2}}+\delta _{H_{1}}+\delta _{H_{2}}$ *and* $\Delta _{G}+\Delta _{H}=\Delta _{G_{1}}+\Delta _{G_{2}}+\Delta _{H_{1}}+\Delta _{H_{2}}$.

### Proof

Let *G* and *H* be the graphs with vertex sets $\{u_{1},u_{2},\ldots, u_{n_{1}(n'_{1}+m'_{1})}\}$ and $\{v_{1},v_{2},\ldots, v_{n_{2}(n'_{2}+m'_{2})}\}$, respectively. The proof is similar to that of Theorem 1 using Lemma (3). □

### Corollary 2

*Let* $G=G_{1}+_{Q}H_{1}$ *and* $H=G_{2}+_{Q}H_{2}$, *then*

- $2\beta (\delta _{G}+\delta _{H} )^{2}\leq F(G\Box H)\leq 2\beta (\Delta _{G}+\Delta _{H} )^{2}$,
- $[2(\delta _{G}+\delta _{H}) ]^{\frac{\beta }{2}}\leq \operatorname{NK}(G\Box H) \leq [2(\Delta _{G}+\Delta _{H}) ]^{\frac{\beta }{2}}$,

*where* $\beta =\frac{1}{2} [n_{1}(n'_{1}+m'_{1}) \{2n_{2} m'_{2}+n'_{2} m_{2}(m_{2}+3) \} +n_{2}({n}'_{2}+m'_{2}) \{2n_{1} m'_{1}+n'_{1} m_{1}(m_{1}+3) \} ]$, $\delta _{G}+\delta _{H}=\delta _{G_{1}}+\delta _{G_{2}}+\delta _{H_{1}}+\delta _{H_{2}}$ *and* $\Delta _{G}+\Delta _{H}=\Delta _{G_{1}}+\Delta _{G_{2}}+\Delta _{H_{1}}+\Delta _{H_{2}}$.

### Proof

(a) By using the relation $F(G)=M_{3}(G)-2 M_{2}(G)$, in Theorem 2, we obtain the required result.

(b) By using the relation $\operatorname{NK}(G)=\sqrt{\operatorname{PM}_{1}(G)}$, in Theorem 2, we obtain the required result. □

In the following theorem we determine the lower and upper bounds for the first Zagreb, ABC, the third Zagreb, the augmented Zagreb, the first multiple Zagreb and GA indices of the Cartesian product of *F*-sum of graphs in terms of their factor graphs for $F=R$.

### Theorem 3

*Let* $G=G_{1}+_{R}H_{1}$ *and* $H=G_{2}+_{R}H_{2}$, *then*

- $2\gamma (\delta_{G}+\delta_{H} )\leq M_{1}(G\Box H) \leq 2\gamma (\Delta_{G}+\Delta_{H} )$,
- $\gamma \frac{\sqrt{2(\delta _{G}+\delta _{H}-1)}}{\Delta _{G}+\Delta _{H}}\leq \operatorname{ABC}(G\Box H)\leq \gamma \frac{\sqrt{2(\Delta _{G}+\Delta _{H}-1)}}{\delta _{G}+\delta _{H}}$.
- $4\gamma (\delta_{G}+\delta_{H} )^{2}\leq M_{3}(G\Box H) \leq 4\gamma (\Delta_{G}+\Delta_{H} )^{2}$,
- $\frac{1}{8}\gamma [\frac{(\delta_{G}+\delta_{H})^{2}}{\Delta _{G}+\Delta_{H}-1} ]^{3}\leq \operatorname{AZI}(G\Box H)\leq \frac{1}{8}\gamma [\frac{(\Delta_{G}+\Delta_{H})^{2}}{\delta_{G}+\delta_{H}-1} ]^{3}$,
- $2^{\gamma } (\delta_{G}+\delta_{H} )^{\gamma }\leq \operatorname{PM}_{1}(G \Box H) \leq 2^{\gamma } (\Delta_{G}+\Delta_{H} )^{\gamma }$,
- $\gamma (\frac{\delta _{G}+\delta _{H}}{\Delta _{G}+\Delta _{H}} )\leq \operatorname{GA}(G\Box H) \leq \gamma (\frac{\Delta _{G}+\Delta _{H}}{\delta _{G}+\delta _{H}} )$,

*where* $\gamma =n_{1}(n'_{1}+m'_{1})(m_{2}n'_{2}+3n_{2}m'_{2}) +n_{2}( {n}'_{2}+m'_{2})(m_{1}n'_{1}+3n_{1}m'_{1})$, $\delta_{G}+\delta_{H}=\delta_{G_{1}}+\delta_{G_{2}}+\delta_{H_{1}}+ \delta_{H_{2}}$ *and* $\Delta_{G}+\Delta_{H}=\Delta_{G_{1}}+\Delta_{G _{2}}+\Delta_{H_{1}}+\Delta_{H_{2}}$.

### Proof

Let *G* and *H* be the graphs with vertex sets $\{u_{1},u_{2},\ldots,u _{n_{1}(n'_{1}+m'_{1})}\}$ and $\{v_{1},v_{2},\ldots, v_{n_{2}(n'_{2}+m'_{2})} \}$, respectively. The proof is similar to that of Theorem 1 using Lemma (4). □

We determine the lower and upper bounds for the *F*-index and the Narumi-Katayama index of the Cartesian product of *F*-sum of graphs in terms of their factor graphs for $F=R$.

### Corollary 3

*Let* $G=G_{1}+_{R}H_{1}$ *and* $H=G_{2}+_{R}H_{2}$, *then*

- $2\gamma (\delta_{G}+\delta_{H} )^{2}\leq F(G\Box H) \leq 2\gamma (\Delta_{G}+\Delta_{H} )^{2}$,
- $[2(\delta_{G}+\delta_{H}) ]^{\frac{\gamma }{2}}\leq \operatorname{NK}(G \Box H) \leq [2(\Delta_{G}+\Delta_{H}) ]^{\frac{\gamma }{2}}$,

*where* $\gamma =n_{1}(n'_{1}+m'_{1})(m_{2}n'_{2}+3n_{2}m'_{2})+n_{2}(n'_{2}+m'_{2})(m _{1}n'_{1}+3n_{1}m'_{1})$, $\delta_{G}+\delta_{H}=\delta_{G_{1}}+\delta_{G_{2}}+\delta_{H_{1}}+ \delta_{H_{2}}$ *and* $\Delta_{G}+\Delta_{H}=\Delta_{G_{1}}+\Delta_{G _{2}}+\Delta_{H_{1}}+\Delta_{H_{2}}$.

### Proof

(a) Using the relation $F(G)=M_{3}(G)-2 M_{2}(G)$, in Theorem 3, we get the required result.

(b) Using the relation $\operatorname{NK}(G)=\sqrt{\operatorname{PM}_{1}(G)}$, in Theorem 3, we get the required result. □

In the following theorem we determine the lower and upper bounds for the first Zagreb, ABC, the third Zagreb, the augmented Zagreb, the first multiple Zagreb and GA indices of the Cartesian product of *F*-sum of graphs in terms of their factor graphs for $F=T$.

### Theorem 4

*Let* $G=G_{1}+_{T}H_{1}$ *and* $H=G_{2}+_{T}H_{2}$, *then*

- $2\eta (\delta _{G}+\delta _{H} )\leq M_{1}(G\Box H)\leq 2\eta (\Delta _{G}+\Delta _{H} )$,
- $\eta \frac{\sqrt{2(\delta _{G}+\delta _{H}-1)}}{\Delta _{G}+\Delta _{H}}\leq \operatorname{ABC}(G\Box H)\leq \eta \frac{\sqrt{2(\Delta _{G}+\Delta _{H}-1)}}{\delta _{G}+\delta _{H}}$,
- $4\eta (\delta _{G}+\delta _{H} )^{2}\leq M_{3}(G\Box H)\leq 4\eta (\Delta _{G}+\Delta _{H} )^{2}$,
- $\frac{1}{8}\eta [\frac{(\delta _{G}+\delta _{H})^{2}}{\Delta _{G}+\Delta _{H}-1} ]^{3}\leq \operatorname{AZI}(G\Box H)\leq \frac{1}{8}\eta [\frac{(\Delta _{G}+\Delta _{H})^{2}}{\delta _{G}+\delta _{H}-1} ]^{3}$,
- $2^{\eta } (\delta _{G}+\delta _{H} )^{\eta }\leq \operatorname{PM}_{1}(G\Box H) \leq 2^{\eta } (\Delta _{G}+\Delta _{H} )^{\eta }$,
- $\eta (\frac{\delta _{G}+\delta _{H}}{\Delta _{G}+\Delta _{H}} )\leq \operatorname{GA}(G\Box H) \leq \eta (\frac{\Delta _{G}+\Delta _{H}}{\delta _{G}+\delta _{H}} )$,

*where* $\eta =\frac{1}{2} [n_{1}(n'_{1}+m'_{1}) \{2n_{2} m'_{2}+n'_{2} m_{2}(m_{2}+5) \} +n_{2}({n}'_{2}+m'_{2}) \{2n_{1} m'_{1}+n'_{1} m_{1}(m_{1}+5) \} ]$, $\delta _{G}+\delta _{H}=\delta _{G_{1}}+\delta _{G_{2}}+\delta _{H_{1}}+\delta _{H_{2}}$ *and* $\Delta _{G}+\Delta _{H}=\Delta _{G_{1}}+\Delta _{G_{2}}+\Delta _{H_{1}}+\Delta _{H_{2}}$.

### Proof

Let *G* and *H* be the graphs with vertex sets $\{u_{1},u_{2},\ldots, u_{n_{1}(n'_{1}+m'_{1})}\}$ and $\{v_{1},v_{2},\ldots, v_{n_{2}(n'_{2}+m'_{2})}\}$, respectively. The proof is similar to that of Theorem 1 using Lemma (5). □

We determine the lower and upper bounds for the *F*-index and the Narumi-Katayama index of the Cartesian product of *F*-sum of graphs in terms of their factor graphs for $F=T$.

### Corollary 4

*Let* $G=G_{1}+_{T}H_{1}$ *and* $H=G_{2}+_{T}H_{2}$, *then*

- $2\eta (\delta _{G}+\delta _{H} )^{2}\leq F(G\Box H)\leq 2\eta (\Delta _{G}+\Delta _{H} )^{2}$,
- $[2(\delta _{G}+\delta _{H}) ]^{\frac{\eta }{2}}\leq \operatorname{NK}(G\Box H) \leq [2(\Delta _{G}+\Delta _{H}) ]^{\frac{\eta }{2}}$,

*where* $\eta =\frac{1}{2} [n_{1}(n'_{1}+m'_{1}) \{2n_{2} m'_{2}+n'_{2} m_{2}(m_{2}+5) \} +n_{2}({n}'_{2}+m'_{2}) \{2n_{1} m'_{1}+n'_{1} m_{1}(m_{1}+5) \} ]$, $\delta _{G}+\delta _{H}=\delta _{G_{1}}+\delta _{G_{2}}+\delta _{H_{1}}+\delta _{H_{2}}$ *and* $\Delta _{G}+\Delta _{H}=\Delta _{G_{1}}+\Delta _{G_{2}}+\Delta _{H_{1}}+\Delta _{H_{2}}$.

### Proof

(a) Using the relation $F(G)=M_{3}(G)-2 M_{2}(G)$, in Theorem 4, we get the required result.

(b) Using the relation $\operatorname{NK}(G)=\sqrt{\operatorname{PM}_{1}(G)}$, in Theorem 4, we get the required result. □

In the following theorem we determine the lower and upper bounds for the first Zagreb, ABC, the third Zagreb, the augmented Zagreb, the first multiple Zagreb and *GA* indices of the Cartesian product of *F*-sum of graphs in terms of their factor graphs for $F=S$ and $F=R$.

### Theorem 5

*Let* $G=G_{1}+_{S}H_{1}$ *and* $H=G_{2}+_{R}H_{2}$, *then*

- $2\xi (\delta_{G}+\delta_{H})\leq M_{1}(G\Box H)\leq 2\xi (\Delta _{G}\Delta_{H})$,
- $\xi \frac{\sqrt{2(\delta_{G}\delta_{H}-1)}}{\Delta_{G}+\Delta _{H}}\leq \operatorname{ABC}(G\Box H)\leq \xi \frac{ \sqrt{2(\Delta_{G}+\Delta_{H}-1)}}{\delta_{G}+\delta_{H}}$,
- $4\xi (\delta_{G}+\delta_{H})^{2}\leq M_{3}(G\Box H)\leq 4\xi ( \Delta_{G}+\Delta_{H})^{2}$,
- $\frac{1}{8}\xi [\frac{(\delta_{G}\delta_{H})^{2}}{\Delta _{G}+\Delta_{H}-1} ]^{3}\leq \operatorname{AZI}(G\Box H)\leq \frac{1}{8}\xi [\frac{(\Delta_{G}+\Delta_{H})^{2}}{\delta_{G}+\delta_{H}-1} ]^{3}$,
- $[2(\delta_{G}+\delta_{H}) ]^{\xi }\leq \operatorname{PM}_{1}(G\Box H) \leq [2(\Delta_{G}+\Delta_{H}) ]^{\xi }$,
- $\xi \frac{\delta_{G}+\delta_{H}}{\Delta_{G}+\Delta_{H}}\leq \operatorname{GA}(G \Box H) \leq \xi \frac{\Delta_{G}+\Delta_{H}}{\delta_{G}+\delta_{H}}$,

*where* $\xi =n_{1}(n'_{1}+m'_{1})(m_{2}n'_{2}+2n_{2}m'_{2}) +n_{2}(n'_{2}+m'_{2})(m _{1}n'_{1}+3n_{1}m'_{1})$, $\delta_{G}+\delta_{H}=\delta_{G_{1}}+ \delta_{G_{2}}+\delta_{H_{1}}+\delta_{H_{2}}$ *and* $\Delta_{G}+\Delta _{H}=\Delta_{G_{1}}+\Delta_{G_{2}}+\Delta_{H_{1}}+\Delta_{H_{2}}$.

### Proof

Let *G* and *H* be the graphs with vertex sets $\{u_{1},u_{2},\ldots,u _{n_{1}(n'_{1}+m'_{1})}\}$ and $\{v_{1},v_{2},\ldots, v_{n_{2}(n'_{2}+m'_{2})} \}$, respectively. The proof is similar to that of 1 with $\vert E(G)\vert =m_{1}n'_{1}+2n_{1}m'_{1}$ and $\vert E(H)\vert =m_{2}n'_{2}+3n_{2}m'_{2}$. □

We determine the lower and upper bounds for the *F*-index and the Narumi-Katayama index of the Cartesian product of *F*-sum of graphs in terms of their factor graphs for $F=S$ and $F=R$.

### Corollary 5

*Let* $G=G_{1}+_{S}H_{1}$ *and* $H=G_{2}+_{R}H_{2}$, *then*

- $2\xi (\delta_{G}\delta_{H} )^{2}\leq F(G\Box H)\leq 2 \xi (\Delta_{G}+\Delta_{H} )^{2}$,
- $[2(\delta_{G}+\delta_{H}) ]^{\frac{\xi }{2}}\leq \operatorname{NK}(G \Box H) \leq [2(\Delta_{G}+\Delta_{H}) ]^{\frac{\xi }{2}}$,

*where* $\xi =n_{1}(n'_{1}+m'_{1})(m_{2}n'_{2}+3n_{2}m'_{2})+n_{2}(n'_{2}+m'_{2})(m _{1}n'_{1}+2n_{1}m'_{1})$, $\delta_{G}+\delta_{H}=\delta_{G_{1}}+ \delta_{G_{2}}+\delta_{H_{1}}+\delta_{H_{2}}$ *and* $\Delta_{G}+\Delta _{H}=\Delta_{G_{1}}+\Delta_{G_{2}}+\Delta_{H_{1}}+\Delta_{H_{2}}$.

### Proof

(a) Using the relation $F(G)=M_{3}(G)-2 M_{2}(G)$, in Theorem 5, we get the required result.

(b) Using the relation $\operatorname{NK}(G)=\sqrt{\operatorname{PM}_{1}(G)}$, in Theorem 5, we get the required result. □

## References

[1] Gutman I, Polansky O (1986). Mathematical Concepts in Organic Chemistry.

[2] Trinajstić N (1992). Chemical Graph Theory.

[3] Gutman I, Trinajstić N (1972). Graph theory and molecular orbitals. Total *π*-electron energy of alternate hydrocarbons. Chem. Phys. Lett..

[4] Gutman I, Ruščić B, Trinajstić N, Wilcox CF (1975). Graph theory and molecular orbitals. XII. Acyclic polyenes. J. Chem. Phys..

[5] Balaban AT, Motoc I, Bonchev D, Makenyan O (1983). Topological indices for structure-activity correlations. Top. Curr. Chem..

[6] Diudea MV (2001). QSPR/QSAR Studies by Molecular Descriptors.

[7] Xu K, Das KC (2012). Zagreb indices and polynomials of $\mathrm{TUHRC}_{4}$ and $\mathrm{TUSC}_{4}\mathrm{C}_{8}$ nanotubes. MATCH Commun. Math. Comput. Chem..

[8] Das KC, Gutman I (2004). Some properties of the second Zagreb index. MATCH Commun. Math. Comput. Chem..

[9] Furtula B, Gutman I, Dehmer M (2103). On structural-sensitivity of degree-based topological indices. Appl. Math. Comput..

[10] Gutman I, Das KC (2004). The first Zagreb index 30 years after. MATCH Commun. Math. Comput. Chem..

[11] Narumi H, Katayama H (1984). Simple topological index, a newly devised index characterizing the topological nature of structural isomers of saturated hydrocarbons. Mem. Fac. Eng., Hokkaido Univ..

[12] Estrada E, Torres L, Rodríguez L, Gutman I (1998). An atom-bond connectivity index: modelling the enthalpy of formation of alkanes. Indian J. Chem. Technol..

[13] Estrada E (2008). Atom-bond connectivity and the energetic of branched alkanes. Chem. Phys. Lett..

[14] Vukičević D, Furtula B (2009). Topological index based on the ratios of geometrical and arithmetical means of end-vertex degrees of edges. J. Math. Chem..

[15] Furtula B, Graovac A, Vukičević D (2010). Augmented Zagreb index. J. Math. Chem..

[16] Ghorbani M, Azimi N (2012). Note on multiple Zagreb indices. Iran. J. Math. Chem..

[17] Shirdel GH, Rezapour H, Sayadi AM (2013). The hyper-Zagreb index of graph operations. Iran. J. Math. Chem..

[18] Furtula B, Gutman I (2015). A forgotten topological index. J. Math. Chem..

[19] Xu JM (2001). Topological Structure and Analysis of Interconnection Networks.

[20] Eliasi M, Taeri B (2009). Four new sums of graphs and their Wiener indices. Discrete Appl. Math..

[21] Deng H, Sarala D, Ayyaswamy SK, Balachandran S (2016). The Zagreb indices of four operations on graphs. Appl. Math. Comput..

[22] Akhter S, Imran M (2016). The sharp bounds on general sum-connectivity index of four operations on graphs. J. Inequal. Appl..

[23] Hayat S, Imran M (2014). Computation of topological indices of certain networks. Appl. Math. Comput..

[24] Hayat S, Imran M (2015). Computation of certain topological indices of nanotubes. J. Comput. Theor. Nanosci..

[25] Hayat S, Siddiqui HMA (2016). On bipartite edge frustration of carbon and boron nanotubes. Stud. Univ. Babeş-Bolyai, Chem..

[26] Imran M, Hayat S, Malik MYH (2014). On topological indices of certain interconnection networks. Appl. Math. Comput..

[27] Li, Y, Yingfang, L, Hayat, S, Siddiqui, HMA, Imran, M, Ahmad, S, Farahani, MR: On degree based and frustration related topological descriptors of single-walled titania nanotubes. J. Comput. Theor. Nanosci. (accepted in press 2016)

